# Supplementary material for: Measuring the experience of social connection within specific social interactions: The Connection During Conversations Scale (CDCS)
Source: PLoS One. 2024 Jan 18;19(1):e0286408. doi: 10.1371/journal.pone.0286408 (PMC10795981; doi:10.1371/journal.pone.0286408)
Supplement: S1 Table — (DOCX) [file pone.0286408.s001.docx]

**S1 Table. List of Connection-Relevant Scales Used in Scale Creation and Other Recent Scales.**

| **Name of Scale** | **Type of Scale** | **Example Item** | **Scaling** | **Citation** | **Subscales** |
| --- | --- | --- | --- | --- | --- |
| ***Scales Used in Scale Creation*** | | | | | |
| Inventory of Socially Supportive Behaviors | Global | Looked after a family member when you were away | 1 (not at all) - 5 (about every day) | Barrera, Sandler, & Ramsay (1981) |  |
| Buckner Neighborhood Cohesion Index | Global | I feel like I belong to this neighborhood | 1 (strongly disagree) - 5 (strongly agree) | Buckner (1988) |  |
| Communal Orientation Scale | Global | It bothers me when other people neglect my needs | 1 (extremely uncharacteristic of them) - 5 (extremely characteristic of them) | Clark, Oullette, Powell, & Milberg (1987) | 3 Subscales: General communal, a desire for other's help, locus of initiation |
| Relational Interdependent Self-Construal | Global | My close relationships are an important reflection of who I am | 1 (very strongly disagree) - 7 (very strongly agree) | Cross, Bacon, & Morris (2000) |  |
| Social Provisions Scale | Global | There are people that I can depend on to help me if I really need it | 1 (Strongly disagree) - 4 (Strongly agree) | Cutrona & Russell (1987) | 6 Subscales: Attachment, social integration, reassurance of worth, reliable alliance, guidance, opportunity for nurturance |
| Interpersonal Reactivity Index | Global | After seeing a play or movie, I have felt as though I were one of the characters | 0 (does not describe me well) - 4 (describes me very well) | Davis (1980) | 4 Subscales: Fantasy items, perspective-taking items, empathic concern items, personal distress items |
| Measurement of Social Disconnection | Global | Today, I generally felt connected to others | 1 (strongly disagree) - 7 (strongly agree) | Eisenberger, Gable, & Lieberman (2007) but adapted from Williams, Cheung, & Choi (2000) |  |
| Facebook Social Connectedness | Global | I feel close to people on Facebook | 1 (strongly agree) - 6 (strongly disagree) | Grieve, Indian, Witteveen, Anne Tolan, & Marrington (2013) |  |
| Sense of Belonging Index | Global | Not sure if I fit with friends | 1 (not relevant) - 4 (very relevant) | Hagerty & Patusky (1995) | 2 Subscales: Psychological state of belonging and antecedents of belonging |
| Four-Dimensional Connectedness Scale | Global | I am appreciated by the people I work with | 1 (strongly disagree) - 7 (strongly agree) | Huynh, Metzer, & Winefield (2012) |  |
| The Social Orientation Scale | Global | It's easy for me to get so caught up in a conversation with my partner that I lose all track of time | 1 (very uncharacteristic of me) - 4 (very characteristic of me) | Ickes, Hutchinson, & Mashek (2004) | 2 Subscales: Social absorption, social individuation |
| The Flow State Scale | Global | I was challenged, but I believed my skills would allow me to meet the challenge | 1 (strongly disagree) - 5 (strongly agree) | Jackson & Marsh (1996) | 9 Subscales: Challenge-skill, action-awareness, clear goals, unambiguous feedback, concentration, sense of control, loss of self-consciousness, Transformation of time, autotelic experience |
| Hemingway Measure of Adolescent Connectedness | Global | Spending time with my friends is the best part of my day | 1 (not at all) - 5 (very true) | Karcher [unpublished] |  |
| Conflict Over Emotional Expression | Global | It is hard to find the right words to indicate to others what I am really feeling | 1 (strongly disagree) - 7 (strongly agree) | King & Emmons (1990) |  |
| Family Allocentricism Scale | Global | I think it is important to get along with my family at all costs | 1 (strongly disagree) - 5 (strongly agree) | Lay, Fairlie, Jackson, Ricci, Eisenberg, Sato, Teeäär, & Melamud (1998) |  |
| Social Connectedness Scale/Social Assurance Scale | Global | I have no sense of togetherness with my peers | 1 (agree) - 6 (disagree) | Lee & Robbins (1995) | 2 Subscales: Social connectedness & social assurance |
| Social Connectedness Scale-Revised | Global | I feel understood by the people I know | 1 (strongly agree) - 6 (strongly disagree) | Lee, Draper, & Lee (2001) | Revising the Social connectedness Scale with 1 subscale |
| Psychological Sense of Community | Global | There is a strong feeling of togetherness on campus | 1 (strongly disagree) - 5 (strongly agree) | Lounsbury & DeNeui (1996) |  |
| Collective Self-Esteem Scale | Global | I am a worthy member of the social groups I belong to | 1 (strongly disagree) - 7 (strongly agree) | Luhtanen & Crocker (1992) | 4 Subscales: Membership, private, public, identity |
| Connectedness With Nature | Global | I think of the natural world as a community to which I belong | 1 (strongly disagree) - 5 (strongly agree) | Mayer & Frantz (2004) |  |
| Self-Disclosure Index | Global | My personal habits | 0 (discuss not at all) - 4 (discuss fully and completely) | Miller, Berg, & Archer (1983) |  |
| Psychological Sense of Community | Global | I have friends in my local neighborhood, who are part of my everyday activities | 1 (very strongly disagree) - 7 (very strongly agree) | Obst, Smith, & Zinkiewicz (2002) | 5 Subscales: Ties and friendship, influence, support, belonging, conscious identification |
| Perceived Social Support from Friends and Family (PSS-Fr and PSS-Fa scales) | Global | My friends/family give me the moral support I need | Yes, No, Don't know | Procidano & Heller (1983) | 2 Subscales: Family, friends |
| ESTCOL Scale | Global | In life, family interests are most important | 1 (Strongly disagree) - 5 (Strongly agree) | Realo, Allik, & Vadi (1997) | 3 Subscales (subtypes of collectivism): Relations with family (familism), friends (companionship), and society (patriotism) |
| Register-Connectedness Scale for Older Adults | Global | Wanted to be with my family | 1 (not important) - 4 (very important) | Register, Herman, & Tavakoli (2011) | 5 Subscales: Self-regulating, facing aging, being part of a family, having friends , being spiritual |
| UCLA Loneliness Scale | Global | I have a lot in common with the people around me | 1 (never) - 4 (often) | Russell, Peplau, & Cutrona (1980) |  |
| Social Support Questionnaire | Global | Who accepts you totally, including both your worst and best points? | Two Parts: (1) Number of available others the individual feels they can turn to; (2) 1 (very dissatisfied) - 6 (very satisfied) | Sarason, Levine, Basham, & Sarason (1983) | 2 Subscales: Perceived availability, satisfaction |
| Emotional Intelligence Scale | Global | I know when to speak about my personal problems to others | 1 (strongly disagree) - 5 (strongly agree) | Schutte, Malouff, Hall, Haggerty, Cooper, Golden & Dornheim (1998) |  |
| Steen Happiness Index - Connection Subscale | Global | I feel disconnected from other people | 1 (Extremely negative) - 5 (Extremely positive) | Seligman, Steen, Park, & Peterson (2005) |  |
| Two-Way Social Support Scale | Global | There is someone I can talk to about the pressures in my life | 0 (not at all) - 5 (always) | Shakespeare-Finch & Obst (2011) | 4 Subscales: Receiving emotional support, giving emotional support, receiving instrumental support, giving instrumental support |
| Balanced Measure of Psychological Needs Scale | Global | I felt close and connected with other people who are important to me | 1 (strongly disagree) - 5 (strongly agree) | Sheldon & Hilpert (2012) |  |
| Self-Construal Scale | Global | My happiness depends on the happiness of those around me | 1 (strongly disagree) - 7 (strongly agree) | Singelis (1994) | 2 Subscales: Interdependent, independent |
| Big Five Inventory (BFI-2) - Extraversion subscale | Global | I am someone who is outgoing, sociable | 1 (disagree strongly) - 5 (agree strongly) | Soto & John (2017) | 5 Subscales: Extraversion, agreeableness, conscientiousness, negative emotionality, open-mindedness |
| Comprehensive Inventory of Thriving - Support, Belonging, Loneliness subscales | Global | Support: There are people that I can depend on to help me Belonging: I feel a sense of belonging in my community.  Loneliness: I feel lonely | 1 (strongly disagree) - 5 (strongly agree) | Su, Tay, & Diener (2014) |  |
| Interpersonal Relationship Index | Global | I can count on a friend | 1 (strongly disagree) - 5 (strongly agree) and 1 (never) - 5 (very often) | Tilden, Nelson, & May (1990) | 3 Subscales: Social support, reciprocity, and conflict |
| Separateness -Connectedness Scale | Global | I often ﬁnd that I can remain cool in spite of people around me being excited | 1 (does not describe me at all) - 5 (describes me very well) | Wang & Mowen (1997) | 2 Subscales: Independence/Individuality, self-other boundary |
| Social Avoidance and Distress Scale | Global | I try to avoid situations which force me to be very sociable | 1 (not at all) - 5 (very much) | Watson & Friend (1969) |  |
| Fear of Negative Evaluation Scale | Global | The opinions that important people have of me cause me little concern | 1 (not at all) - 5 (very much) | Watson & Friend (1969) |  |
| Multi-Dimensional Support Scale | Global | How often did they really listen to you when you talked about your concerns or problems? | 1 (never) - 4 (often); Would have liked: more, less, right | Winefield, Winefield, & Tiggemann (1992) |  |
| Multi-Dimensional Scale of Perceived Social Support | Global | I get the emotional help and support I need from my family | 1 (very strongly disagree) - 7 (very strongly agree) | Zimet, Dahlem, Zimet, & Farley (1988) |  |
| Inclusion of Others in Self Scale | Partner-Specific | Increasingly overlapping circles |  | Aron, Aron, & Smollan (1992) |  |
| Individuality and Connectedness Q-Sort | Partner-Specific | Speaks first; Initiate’s compromise; Asks for partner's opinion | Q-Sort | Bengtson & Grotevant (1999) |  |
| Relationship Closeness Inventory | Partner-Specific | ______ influences important things in my life | 1 (very strongly disagree) - 7 (very strongly agree) | Berscheid, Snyder, & Omoto (1989) |  |
| Fear of Intimacy Scale | Partner-Specific | I would feel uneasy about talking with _____ about something that has hurt me deeply | 1 (not at all characteristic of me) - 5 (extremely characteristic of me) | Descutner & Thelen (1991) |  |
| The Group Attitude Scale | Partner-Specific | I feel included in the group | 1 (disagree) - 9 (agree) | Evans & Jarvis (1986) |  |
| Relationship Attributions Scale | Partner-Specific | Your partner criticizes something you say | 1 (strongly disagree) - 7 (strongly agree) | Fincham & Bradbury (1992) | 2 Subscales: Causal-attribution, responsibility-attribution |
| The Experiences in Close Relationships-Relationship Structures Questionnaire | Partner-Specific | I usually discuss my problems and concerns with this person | 1 (strongly disagree) - 7 (strongly agree) | Fraley, Heffernan, Vicary, & Brumbaugh (2011) | 4 Subscales: Mother, father, romantic partners, best friends |
| Couples Satisfaction Index | Partner-Specific | I have a warm and comfortable relationship with my partner | 1 (strongly disagree) - 6 (strongly agree) | Funk & Rogge (2007) |  |
| Passionate Love Scale | Partner-Specific | Since I've been involved with ______, my emotions have been on a roller coaster | 1 (Not at all true) - 9 (Definitely true) | Hatfield & Sprecher (1986) |  |
| Relationship Assessment Scale | Partner-Specific | How well does your partner meet your needs? | 1 (low satisfaction) - 5 (high satisfaction) | Hendrick, Dicke, & Hendrick (1998) |  |
| Commitment Scale | Partner-Specific | How likely is it that your relationship will be permanent? | 7-pt scale | Lund (1985) |  |
| Investment Scale | Partner-Specific | Spending your free time with your partner rather than doing other things or seeing other people. | How large an investment on 7-pt scale | Lund (1985) |  |
| Inclusion of Community in Self Scale | Partner-Specific | Increasingly overlapping circles |  | Mashek, Cannaday, & Tangney (2007) |  |
| Miller Social Intimacy Scale | Partner-Specific | How much do you like to spend time alone with him/her? | 1 (very rarely) - 10 (almost always) | Miller & Lefcourt (1982) |  |
| Measurement of Communal Strength | Partner-Specific | How happy do you feel when doing something that helps ____? | 0 (not at all) - 10 (extremely) | Mills, Clark, Ford, & Johnson (2004) |  |
| Partner Responsiveness Scale | Partner-Specific | ...sees the "real" me | 1 (strongly disagree) - 7 (strongly agree) | Reis, Maniaci, Caprariello, Eastwick, & Finkel (2011); Reis et al. (2017) |  |
| Rubin's Loving and Liking Scale | Partner-Specific | Love: I feel that I can confide in ____ about virtually everything Like: When I am with ______, we are almost always in the same mood | 1 (not at all true; disagree completely) - 9 (definitely true; agree completely) | Rubin (1970) |  |
| Personal Assessment of Intimacy in Relationships Inventory | Partner-Specific | I think that we share some of the same interests | 1 (strongly disagree) - 5 (strongly agree) | Schaefer & Olson (1981) | 6 Subscales: Emotional intimacy, social intimacy, sexual intimacy, intellectual intimacy, recreational intimacy, conventionality |
| Revised Experiences in Close Relationships Questionnaire | Partner-Specific | I often worry that my partner will not want to stay with me | 1 (strongly disagree) - 6 (strongly agree) | Sibley, Fischer, & Liu (2005) | 2 Subscales: Romantic attachment anxiety and romantic attachment avoidance |
| Dyadic Adjustment Scale | Partner-Specific | Amount of time spent together | 0 (always disagree) - 5 (always agree) | Spanier (1976) | 4 Subscales: Dyadic consensus, dyadic satisfaction, dyadic cohesion, affectional expression |
| Sternberg Intimacy Scale | Partner-Specific | I am able to count on ____ in times of need | 1 (not at all) - 9 (extremely) | Sternberg (1997) | 3 Subscales: Intimacy, passion, commitment |
| Positivity Resonance Scale | Interaction-Specific | Did you experience a mutual sense of warmth and concern toward the other(s)? | 0 - 100% | Major, Nguyen, Lundberg, & Fredrickson (2018) |  |
| **Name of Scale** | **Type of Scale** | **Example Item** | **Scaling** | **Citation** | **Subscales** |
| ***Recent Scales Not Used in Scale Creation*** | | | | | |
| Friendship Network Satisfaction | Global | My friends understand me | 0 (Not all agree) - 5 (Completely Agree) | Kaufman, Perez, Reise, Bradbury, & Karney (2021) | 2 Subscales: Closeness and socializing |
| Generalized Shared Reality Measure - Cross Situational | Partner-Specific | We frequently think of things at the exact same time | 1 (strongly disagree) - 7 (strongly agree) | Rossignac-Milon, Bolger, Zee, Boothby, & Higgins (2021) |  |
| Generalized Shared Reality Measure - Interaction-Specific | Interaction-Specific | During our interaction... we thought of things at the exact same time | 1 (strongly disagree) - 7 (strongly agree) | Rossignac-Milon, Bolger, Zee, Boothby, & Higgins (2021) |  |
