# Supplementary material for: Measuring the experience of social connection within specific social interactions: The Connection During Conversations Scale (CDCS)
Source: PLoS One. 2024 Jan 18;19(1):e0286408. doi: 10.1371/journal.pone.0286408 (PMC10795981; doi:10.1371/journal.pone.0286408)
Supplement: S2 Table — Note. SR = Shared Reality Subscale. PR = Partner Responsiveness Subscale. PI = Participant Interest Subscale. AE = Affective Experience subscale. * p < .05. ** p < .01. *** p < .001. (DOCX) [file pone.0286408.s002.docx]

**S2 Table. Correlations among the Connection During Conversations Scale (CDCS) Items across three occasions (Times 1, 2, and 3) in Study 2**

| Sub-scale | Item | Correlations between Time 1 and Time 2 | Correlations between Time 2 and Time 3 | Correlations between Time 1 and Time 3 |
| --- | --- | --- | --- | --- |
| SR | 1. I felt “in sync” with them | .16** | .27*** | .17** |
| SR | 2. I felt like we shared a lot in common | .20*** | .27*** | .18** |
| SR | 3. I felt that we saw the world in the same way | .15** | .25*** | .06 |
| SR | 4. They were able to relate to my experiences | .14* | .14* | .13* |
| PR | 5. They were interested in my thoughts and feelings | .27*** | .30*** | .23*** |
| PR | 6. They respected my beliefs and opinions | .28*** | .21*** | .13* |
| PR | 7. I felt that they cared about me | .23*** | .20*** | .13* |
| PR | 8. They really understood who I am | .23*** | .28*** | .20*** |
| PI | 9. I was truly attentive during the interaction | .23*** | .29*** | .21*** |
| PI | 10. I was interested in their thoughts and feelings | .24*** | .27*** | .23*** |
| PI | 11. I thought that they were boring | .23*** | .24*** | .26*** |
| PI | 12. I was distracted during the conversation (R) | .21*** | .36*** | .28*** |
| AE | 13. I was nervous during the interaction (R) | .12* | .30*** | .29*** |
| AE | 14. I felt that my energy was drained by the interaction (R) | .32*** | .20*** | .17** |
| AE | 15. I couldn’t wait for the interaction to end (R) | .27*** | .20*** | .25*** |
| AE | 16. I felt that it was hard to communicate with them (R) | .23*** | .24*** | .26*** |

*Note.* SR = Shared Reality subscale. PR = Partner Responsiveness subscale. PI = Participant Interest subscale. AE = Affective Experience subscale. **p* < .05. ** *p* < .01. *** *p* < .001.
